# The association between non-alcoholic fatty liver disease and atopic dermatitis: a population-based cohort study

**DOI:** 10.3389/fimmu.2023.1171804

**Published:** 2023-08-18

**Authors:** Shuo-Yan Gau, Ching-Hua Huang, Yih Yang, Tung-Han Tsai, Kuang-Hua Huang, Chien-Ying Lee

**Affiliations:** ^1^ School of Medicine, Chung Shan Medical University, Taichung, Taiwan; ^2^ Department of Pharmacy, Chung Shan Medical University Hospital, Taichung, Taiwan; ^3^ Department of Pharmacology, Chung Shan Medical University, Taichung, Taiwan; ^4^ Institute of Medicine, Chung Shan Medical University, Taichung, Taiwan; ^5^ Department of Obstetrics and Gynecology, E-Da Hospital, I-Shou University, Kaohsiung, Taiwan; ^6^ Department of Health Services Administration, China Medical University, Taichung, Taiwan

**Keywords:** non-alcoholic fatty liver disease, atopic dermatitis, NHIRD, cohort study, epidemiology

## Abstract

**Background:**

In previous studies, it was reported that non-alcoholic fatty liver disease (NAFLD) incidence and prevalence increased in children with atopic dermatitis. Nevertheless, the actual association between the two diseases has not been fully proven in large-scale studies, and real-world evidence is missing. The objective of this nationwide, longitudinal cohort study was to evaluate the association between NAFLD and atopic dermatitis.

**Methods:**

The National Health Insurance Research Database in Taiwan was utilized in this study. Patients with records of NAFLD diagnosis were recruited as the experimental group, and patients having less than three outpatient visits or one inpatient visiting record due to NAFLD were excluded from the study design. Non-NAFLD controls were matched based on a 1:4 propensity score matching. Potential confounders including age, gender, comorbidity, and medical utilization status were considered as covariates. The risk of future atopic dermatitis would be evaluated based on multivariate Cox proportional hazard regression.

**Results:**

Compared with people without NAFLD, a decreased risk of atopic dermatitis in NALFD patients had been observed (aHR = 0.93, 95% CI 0.87–0.98). The trend was especially presented in young NAFLD patients. In patients younger than 40 years old, a 20% decreased risk of atopic dermatitis was reported (aHR = 0.80, 95% CI 0.70–0.92).

**Conclusion:**

People with NAFLD were not associated with an increased risk of atopic dermatitis. Conversely, a 0.93-fold risk was noted in NAFLD patients, compared with NAFLD-free controls. Future studies are warranted to evaluate further the mechanism regarding the interplay between the inflammatory mechanisms of NAFLD and atopic dermatitis.

## Introduction

With increasing prevalence, non-alcoholic fatty liver disease (NAFLD) causes a gradually increasing health burden worldwide (1). The prevalence of NAFLD varies in different regions. In a recent meta-analysis, the global prevalence was reported to be approximately 25% (2). NAFLD was strongly associated with an increased risk of liver cirrhosis and hepatocellular carcinoma, leading to worsened prognosis and increased mortality rate (2, 3). The influence of NAFLD on health status was not limited to hepatic dysfunction. Multisystem comorbidities, including cardiovascular, renal, and endocrinological dysfunctions, were reported to be presented in NAFLD patients (4).

Patients with atopic dermatitis present pruritus and skin lesions at various sites (5). Environmental factors such as climate, diet, and chemical exposure could contribute to the onset of atopic dermatitis (6, 7). Moreover, factors including antibiotic use and comorbidity status could also serve as potential risk factors of atopic dermatitis (6, 8). In previous studies, it was reported that NAFLD incidence and prevalence increased in children with atopic dermatitis (9, 10). Nevertheless, the actual association has not been fully proven in large-scale studies.

Evaluating the risk of atopic dermatitis in NAFLD patients could be potentially useful serving as references for clinicians caring for NAFLD patients. However, studies evaluating the risk of atopic dermatitis in NAFLD patients were not available, and real-world evidence is missing. Therefore, we conducted a nationwide, longitudinal cohort study to evaluate the association between NAFLD and atopic dermatitis.

## Material and methods

### Data source

The data source of this retrospective cohort study was based on the datasets from the National Health Insurance Research Database (NHIRD) in Taiwan. Data in NHIRD were recorded based on medical claims and related information retrieved from the mandatory, single-payer National Health Insurance (NHI) program. The NHI covered most of the citizens (>99%) in Taiwan, which could be representative of the population. The NHIRD has been widely utilized as the data source in previous real-world observational studies (11, 12). Deidentified information including inpatient/outpatient visits and diagnosis was recorded in the NHIRD. The *International Classification of Disease, 9th and 10th revision, Clinical Modification* (*ICD-9-CM, ICD-10-CM*) codes were applied for disease recording. Since information that could identify any specific individual has been deidentified, informed consent for the current study was exempted.

### Study population, exposure, and outcomes

Data from 2000 to 2018 in NHIRD have been extracted for analysis. Patients with NAFLD were recruited as the NAFLD group. In the NHIRD, diagnosis was made after evaluations from specialists. However, several additional criteria were set to ensure validity and quality for disease definition: 1) Only patients who were diagnosed with NAFLD between 2004 and 2013 with the index date matching the control group were included in the cohort. In this case, we could ensure that each analyzed patient could have at least a 5-year term for follow-up. 2) To ensure the validity of participant recruitment, patients having less than three outpatient visits or one inpatient visiting record due to NAFLD were excluded from the study design (13). 3) Patients younger than 18 years old were excluded from the study. 4) People who withdrew from the NHIRD or were deceased before the endpoint were censored and were not included for further analysis. 5) To ensure that the outcome measurement could represent new-onset atopic dermatitis, patients with a previous record of atopic dermatitis were excluded from the study. 6) To avoid the presence of outcomes being influenced by liver diseases other than NAFLD, patients with a previous record of chronic liver diseases (including liver fibrosis, cirrhosis, and hepatic cancer) before the index date were excluded from the study. Healthy control groups without NAFLD were randomly selected. Propensity score matching (PSM) in age, sex, income level, urbanization, comorbidity burden, and enrolled year was performed to address the differences in baseline characteristics. After PSM, each patient in the NAFLD group was randomly matched with four NAFLD-free healthy people in the control group. To address possible confounding bias, multivariate Cox regression analysis was performed to adjust variables including related comorbidities such as hypertension, diabetes, hyperlipidemia, myocardial infarction, coronary artery disease, chronic kidney disease, obesity, alcoholism, major depressive disorder, inflammatory bowel disease, asthma, psoriasis, allergic rhinitis, conjunctivitis, and urticaria. The study endpoint was when the study participants were diagnosed with atopic dermatitis. To improve the validity of atopic dermatitis diagnosis, only events consisting of more than three outpatient visits or one inpatient record due to atopic dermatitis were defined as outcome events (14). Each person in the study was followed up from the index date to the date of diagnosis of atopic dermatitis (outcome event), quitting registration from the NHI or on 31 December 2018 (the endpoint of the current study).

### Statistical analysis

*p*-values smaller than 0.05 are considered to be significant differences between the data for comparison. Standardized mean difference (SMD) has been utilized to evaluate the difference between the NAFLD group and the healthy control group regarding baseline characteristics. SMD values smaller than 0.1 represent a negligible difference. To evaluate the risk of incident outcome events, the hazard ratio (HR) was calculated. Each HR was presented with a 95% confidence interval (CI). All statistical analyses were performed by SAS software version 9.4 (SAS Institute, Cary, NC, USA).

## Results

### Baseline characteristics

**Figure 1:**
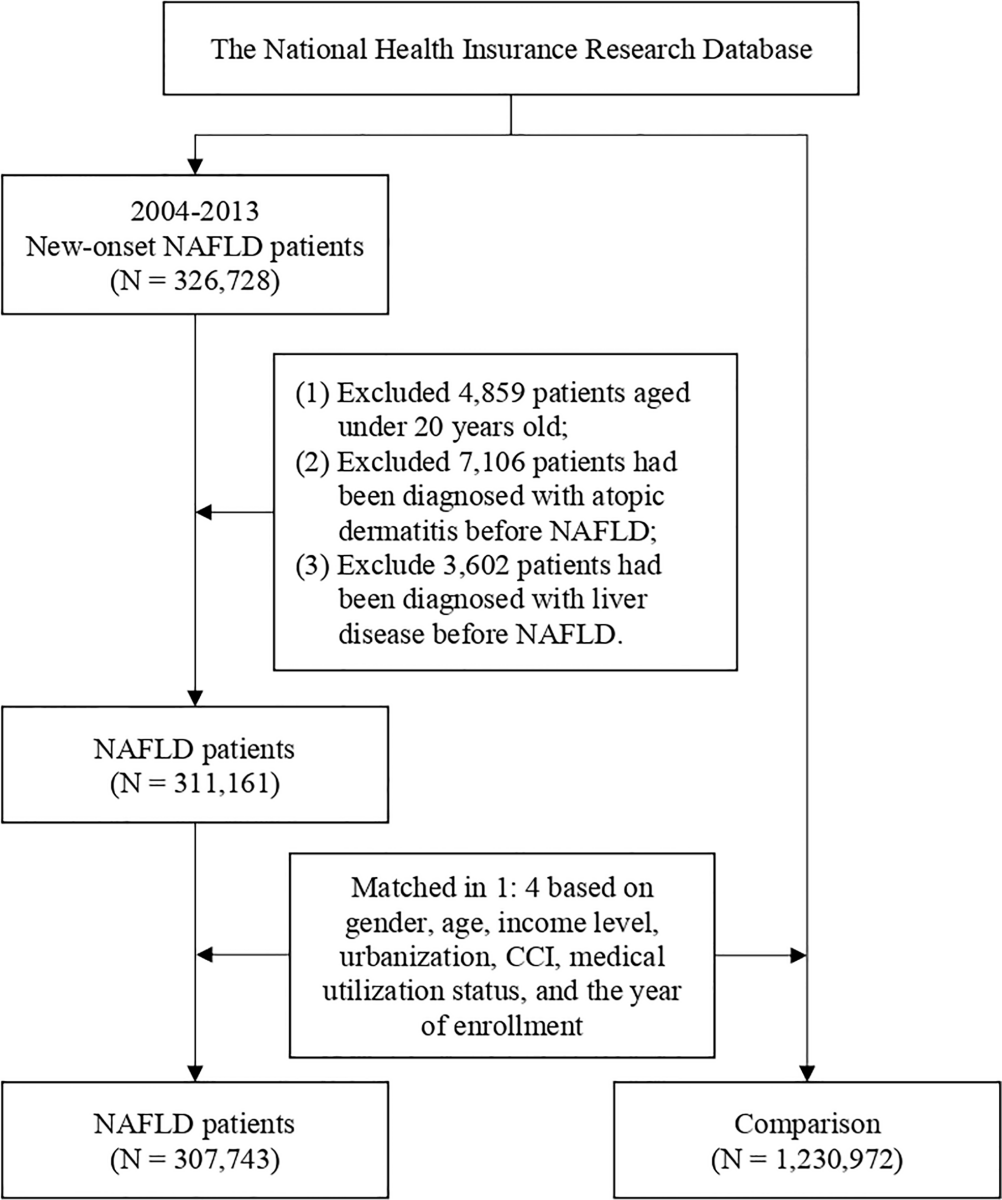
Flowchart diagram of patient selection.

In total, 307,743 patients with NAFLD and 1,230,972 healthy controls were enrolled in the current study (**Figure 1**). In the NAFLD group, 42% of the patients were men and 58% of the patients were women. The mean age of NAFLD patients was 51.2 years old. Comorbidity burden has been evaluated via the Charlson comorbidity index (CCI), a validated assessment tool used to evaluate the burden of comorbidities and to predict patients’ prognostic factors, which has been utilized in previous real-world studies (13, 15). After propensity score matching, differences between related variates become statistically insignificant (**Table 1**).

**Table 1:** Baseline characteristics of patients with non-alcoholic fatty liver disease after matching.

Variables	Total		Non-NAFLD controls		NAFLD		*p*-value^a^	SMD
	N	%	N	%	N	%		
Total	1,538,715	100.00	1,230,972	100.00	307,743	100.00		
Gender^b^							0.537	0.000
Female	651,252	42.32	521,153	42.34	130,099	42.28		
Male	887,463	57.68	709,819	57.66	177,644	57.72		
Age (years)^b^							0.095	0.000
≤40	376,461	24.47	300,706	24.43	75,755	24.62		
41–64	868,775	56.46	695,380	56.49	173,395	56.34		
≥65	293,479	19.07	234,886	19.08	58,593	19.04		
Mean ± SD	51.23 ± 15.94		51.35 ± 16.15		50.75 ± 15.03			
Income level^b^							0.793	0.000
Low income (≤21,000)	732,197	47.58	585,620	47.57	146,577	47.63		
Middle income (21,001–33,000)	355,207	23.08	284,156	23.08	71,051	23.09		
High income (≥33,001)	451,311	29.33	361,196	29.34	90,115	29.28		
Urbanization^b^							0.946	0.000
Level 1	454,408	29.53	363,624	29.54	90,784	29.50		
Level 2	494,332	32.13	395,393	32.12	98,939	32.15		
Level 3	236,468	15.37	189,263	15.38	47,205	15.34		
Level 4	205,783	13.37	164,675	13.38	41,108	13.36		
Level 5	28,429	1.85	22,693	1.84	5,736	1.86		
Level 6	60,436	3.93	48,287	3.92	12,149	3.95		
Level 7	58,859	3.83	47,037	3.82	11,822	3.84		
CCI score^b^							0.937	0.000
0	375,749	24.42	300,597	24.42	75,152	24.42		
1	493,978	32.10	395,253	32.11	98,725	32.08		
2	279,953	18.19	223,844	18.18	56,109	18.23		
≥3	389,035	25.28	311,278	25.29	77,757	25.27		
Length of hospital stay^b^							0.971	0.000
0 days	766,741	49.83	613,395	49.83	153,346	49.83		
1~6 days	394,544	25.64	315,677	25.64	78,867	25.63		
≥7 days	377,430	24.53	301,900	24.53	75,530	24.54		
Enrolled year^b^							1.000	0.000
2004	155,045	10.08	124,036	10.08	31,009	10.08		
2005	141,950	9.23	113,560	9.23	28,390	9.23		
2006	148,475	9.65	118,780	9.65	29,695	9.65		
2007	151,540	9.85	121,232	9.85	30,308	9.85		
2008	150,780	9.80	120,624	9.80	30,156	9.80		
2009	154,980	10.07	123,984	10.07	30,996	10.07		
2010	157,335	10.23	125,868	10.23	31,467	10.23		
2011	154,255	10.02	123,404	10.02	30,851	10.02		
2012	161,885	10.52	129,508	10.52	32,377	10.52		
2013	162,470	10.56	129,976	10.56	32,494	10.56		
Hypertension							<0.001	0.040
No	1,065,223	69.23	856,805	69.60	208,418	67.72		
Yes	473,492	30.77	374,167	30.40	99,325	32.28		
Diabetes							<0.001	0.030
No	1,249,787	81.22	996,513	80.95	253,274	82.30		
Yes	288,928	18.78	234,459	19.05	54,469	17.70		
Hyperlipidemia							<0.001	0.150
No	1,250,340	81.26	1,015,517	82.50	234,823	76.30		
Yes	288,375	18.74	215,455	17.50	72,920	23.70		
Myocardial infarction							<0.001	0.060
No	1,529,486	99.40	1,222,613	99.32	306,873	99.72		
Yes	9,229	0.60	8,359	0.68	870	0.28		
Coronary artery disease							<0.001	0.010
No	1,344,983	87.41	1,076,831	87.48	268,152	87.14		
Yes	193,732	12.59	154,141	12.52	39,591	12.86		
Chronic kidney disease							<0.001	0.120
No	1,501,787	97.60	1,197,486	97.28	304,301	98.88		
Yes	36,928	2.40	33,486	2.72	3,442	1.12		
Obesity							<0.001	0.110
No	1,528,446	99.33	1,225,409	99.55	303,037	98.47		
Yes	10,269	0.67	5,563	0.45	4,706	1.53		
Alcoholism							<0.001	0.030
No	1,530,379	99.46	1,224,978	99.51	305,401	99.24		
Yes	8,336	0.54	5,994	0.49	2,342	0.76		
Inflammatory bowel disease							0.007	0.010
No	1,531,767	99.55	1,225,503	99.56	306,264	99.52		
Yes	6,948	0.45	5,469	0.44	1,479	0.48		
Asthma							<0.001	0.050
No	1,457,748	94.74	1,163,630	94.53	294,118	95.57		
Yes	80,967	5.26	67,342	5.47	13,625	4.43		
Psoriasis							<0.001	0.010
No	1,532,667	99.61	1,226,346	99.62	306,321	99.54		
Yes	6,048	0.39	4,626	0.38	1,422	0.46		
Allergic rhinitis							0.612	0.000
No	1,429,423	92.90	1,143,603	92.90	285,820	92.88		
Yes	109,292	7.10	87,369	7.10	21,923	7.12		
Conjunctivitis							<0.001	0.010
No	1,476,026	95.93	1,181,405	95.97	294,621	95.74		
Yes	62,689	4.07	49,567	4.03	13,122	4.26		
Urticaria							<0.001	0.020
No	1,500,066	97.49	1,200,677	97.54	299,389	97.29		
Yes	38,649	2.51	30,295	2.46	8,354	2.71		

SMD, standardized mean difference.

a Chi-square test.

b Matching variables.

### Risk of NAFLD patients developing a topic dermatitis

For patients with NAFLD, the incidence rate of new-onset atopic dermatitis was 0.37 per 1,000 person-years, whereas for healthy controls without NAFLD, the incidence rate of atopic dermatitis was 0.39 per 1,000 person-years (**Table 2**). In the Cox regression model, compared with healthy controls without NAFLD, people with NALFD had a statistically significant lower risk of developing atopic dermatitis (HR = 0.94, 95% CI 0.88–0.99). The significance remained after adjusting related covariates (aHR = 0.93, 95% CI 0.87–0.98). For patients with inflammatory or allergic comorbidities, including psoriasis, allergic rhinitis, and urticaria, the risk of atopic dermatitis was significantly higher than in controls (**Table 3**).

**Table 2:** Incident atopic dermatitis.

Variables	Incident atopic dermatitis						
	No		Yes		Follow-up (person-years)	IR	IRR (95% CI)
	N	%	N	%			
Total	1,532,865	99.62	5,850	0.38			
Patients							
Non-NAFLD controls	1,226,230	99.61	4,742	0.39	12,139,815	0.39	
NAFLD	306,635	99.64	1,108	0.36	3,030,613	0.37	0.95 (0.89–1.00)
Gender							
Female	648,838	99.63	2,414	0.37	6,353,492	0.38	
Male	884,027	99.61	3,436	0.39	8,816,936	0.39	1.03 (0.97–1.08)
Age (years)							
≤40	374,970	99.60	1,491	0.40	3,740,698	0.40	
41–64	865,789	99.66	2,986	0.34	8,500,235	0.35	0.88 (0.83–0.94)
≥65	292,106	99.53	1,373	0.47	2,929,495	0.47	1.18 (1.10–1.27)
Mean ± SD	51.23 ± 15.93		51.63 ± 17.00				
Income level							
Low income (≤21,000)	728,958	99.56	3,239	0.44	7,725,478	0.42	
Middle income (21,001–33,000)	354,111	99.69	1,096	0.31	3,109,340	0.35	0.83 (0.77–0.88)
High income (≥33,001)	449,796	99.66	1,515	0.34	4,335,611	0.35	0.83 (0.79–0.88)
Urbanization							
Level 1	452,865	99.66	1,543	0.34	4,495,144	0.34	
Level 2	492,477	99.62	1,855	0.38	4,867,138	0.38	1.12 (1.05–1.20)
Level 3	235,565	99.62	903	0.38	2,314,371	0.39	1.15 (1.06–1.25)
Level 4	204,900	99.57	883	0.43	2,024,159	0.44	1.29 (1.19–1.40)
Level 5	28,337	99.68	92	0.32	282,152	0.33	0.97 (0.79–1.20)
Level 6	60,175	99.57	261	0.43	605,162	0.43	1.26 (1.10–1.44)
Level 7	58,546	99.47	313	0.53	582,302	0.54	1.59 (1.41–1.80)
CCI score							
0	374,633	99.70	1,116	0.30	3,618,553	0.31	
1	492,127	99.63	1,851	0.37	4,860,999	0.38	1.23 (1.14–1.33)
2	278,741	99.57	1,212	0.43	2,797,198	0.43	1.39 (1.28–1.52)
≥3	387,364	99.57	1,671	0.43	3,893,679	0.43	1.39 (1.28–1.50)
Length of hospital stay							
0 days	764,032	99.65	2,709	0.35	7,423,457	0.36	
1~6 days	392,932	99.59	1,612	0.41	3,945,567	0.41	1.14 (1.07–1.21)
≥7 days	375,901	99.59	1,529	0.41	3,801,404	0.40	1.11 (1.04–1.18)
Hypertension							
No	1,061,252	99.63	3,971	0.37	10,560,849	0.38	
Yes	471,613	99.60	1,879	0.40	4,609,579	0.41	1.08 (1.03–1.14)
Diabetes							
No	1,245,161	99.63	4,626	0.37	12,362,311	0.37	
Yes	287,704	99.58	1,224	0.42	2,808,117	0.44	1.19 (1.12–1.27)
Hyperlipidemia							
No	1,245,663	99.63	4,677	0.37	12,442,659	0.38	
Yes	287,202	99.59	1,173	0.41	2,727,769	0.43	1.13 (1.06–1.21)
Myocardial infarction							
No	1,523,674	99.62	5,812	0.38	15,080,904	0.39	
Yes	9,191	99.59	38	0.41	89,524	0.42	1.08 (0.82–1.53)
Coronary artery disease							
No	1,339,994	99.63	4,989	0.37	13,221,147	0.38	
Yes	192,871	99.56	861	0.44	1,949,281	0.44	1.16 (1.07–1.25)
Chronic kidney disease							
No	1,496,086	99.62	5,701	0.38	14,822,530	0.38	
Yes	36,779	99.60	149	0.40	347,898	0.43	1.13 (0.97–1.32)
Obesity							
No	1,522,642	99.62	5,804	0.38	15,071,810	0.39	
Yes	10,223	99.55	46	0.45	98,618	0.47	1.21 (0.91–1.61)
Alcoholism							
No	1,524,558	99.62	5,821	0.38	15,086,588	0.39	
Yes	8,307	99.65	29	0.35	83,840	0.35	0.9 (0.62–1.30)
Inflammatory bowel disease							
No	1,525,949	99.62	5,818	0.38	15,101,898	0.39	
Yes	6,916	99.54	32	0.46	68,530	0.47	1.21 (0.86–1.72)
Asthma							
No	1,452,273	99.62	5,475	0.38	14,368,290	0.38	
Yes	80,592	99.54	375	0.46	802,138	0.47	1.24 (1.12–1.38)
Psoriasis							
No	1,526,869	99.62	5,798	0.38	15,112,150	0.38	
Yes	5,996	99.14	52	0.86	58,278	0.89	2.34 (1.79–3.06)
Allergic rhinitis							
No	1,424,134	99.63	5,289	0.37	14,139,934	0.37	
Yes	108,731	99.49	561	0.51	1,030,494	0.54	1.46 (1.34–1.59)
Conjunctivitis							
No	1,470,480	99.62	5,546	0.38	14,548,815	0.38	
Yes	62,385	99.52	304	0.48	621,613	0.49	1.29 (1.15–1.45)
Urticaria							
No	1,494,573	99.63	5,493	0.37	14,790,915	0.37	
Yes	38,292	99.08	357	0.92	379,513	0.94	2.54 (2.28–2.83)

IR, the incidence rate of per 1,000 person-years; IRR, incidence rate ratio.

**Table 3:** The Cox model.

Variables	Unadjusted model^a^			Adjusted model^b^			Competing risk model^c^		
	HR	95% CI	*p*-value	HR	95% CI	*p*-value	HR	95% CI	*p*-value
Patients									
Non-NAFLD controls (ref.)	1			1			1		
NAFLD	0.94	0.88–0.99	0.046	0.93	0.87–0.98	0.024	0.93	0.87–0.99	0.022
Gender									
Female (ref.)	1			1			1		
Male	1.04	0.98–1.09	0.182	1.05	0.99–1.11	0.094	1.07	1.01–1.13	0.021
Age (years)									
≤40 (ref.)	1			1			1		
41–64	0.87	0.82–0.93	<0.001	0.85	0.79–0.90	<0.001	0.85	0.79–0.90	<0.001
≥65	1.18	1.10–1.27	<0.001	1.04	0.96–1.14	0.345	1.03	0.94–1.12	0.517
Income level									
Low income (≤21,000) (ref.)	1			1			1		
Middle income (21,001–33,000)	0.77	0.71–0.82	<0.001	0.98	0.91–1.05	0.525	0.80	0.75–0.86	<0.001
High income (≥33,001)	0.79	0.75–0.84	<0.001	0.94	0.88–1.01	0.081	0.86	0.81–0.92	<0.001
Urbanization									
Level 1 (ref.)	1			1			1		
Level 2	1.11	1.04–1.19	0.003	1.10	1.03–1.18	0.007	1.08	1.01–1.16	0.020
Level 3	1.13	1.04–1.23	0.003	1.12	1.03–1.21	0.008	1.09	1.01–1.19	0.037
Level 4	1.27	1.17–1.38	<0.001	1.22	1.12–1.32	<0.001	1.18	1.08–1.28	0.000
Level 5	0.95	0.77–1.18	0.652	0.87	0.70–1.07	0.195	0.84	0.68–1.03	0.098
Level 6	1.27	1.11–1.45	0.000	1.17	1.02–1.33	0.025	1.13	0.99–1.29	0.070
Level 7	1.57	1.39–1.78	<0.001	1.47	1.30–1.67	<0.001	1.41	1.25–1.60	<0.001
CCI score									
0 (ref.)	1			1			1		
1	1.25	1.16–1.35	<0.001	1.18	1.09–1.27	<0.001	1.21	1.13–1.31	<0.001
2	1.44	1.32–1.56	<0.001	1.29	1.18–1.40	<0.001	1.36	1.24–1.48	<0.001
≥3	1.43	1.32–1.54	<0.001	1.24	1.13–1.35	<0.001	1.31	1.20–1.43	<0.001
Length of hospital stay									
0 days (ref.)	1			1			1		
1~6 days	1.14	1.07–1.21	<0.001	1.03	0.96–1.09	0.405	1.05	0.99–1.12	0.118
≥7 days	1.13	1.06–1.20	<0.001	0.96	0.90–1.03	0.245	0.98	0.92–1.05	0.585
Comorbidities [yes vs. no (ref.)]									
Hypertension	1.07	1.02–1.13	0.011	0.95	0.89–1.01	0.092	0.92	0.87–0.99	0.017
Diabetes	1.15	1.08–1.23	<0.001	1.07	0.99–1.15	0.086	1.05	0.97–1.12	0.217
Hyperlipidemia	1.11	1.04–1.19	0.001	1.11	1.04–1.20	0.003	1.06	0.99–1.14	0.090
Myocardial infarction	1.10	0.80–1.51	0.566	0.97	0.70–1.35	0.872	0.94	0.68–1.30	0.713
Coronary artery disease	1.19	1.10–1.28	<0.001	1.03	0.95–1.11	0.510	1.06	0.98–1.15	0.156
Chronic kidney disease	1.09	0.93–1.28	0.302	1.03	0.87–1.22	0.734	0.96	0.82–1.14	0.661
Obesity	1.20	0.90–1.60	0.227	1.19	0.89–1.60	0.235	1.18	0.89–1.59	0.256
Alcoholism	0.91	0.63–1.31	0.616	0.85	0.59–1.23	0.395	0.83	0.57–1.20	0.318
Inflammatory bowel disease	1.21	0.86–1.72	0.275	1.13	0.80–1.59	0.507	1.12	0.79–1.59	0.510
Asthma	1.23	1.11–1.37	<0.001	1.00	0.90–1.12	0.962	1.01	0.90–1.12	0.896
Psoriasis	2.31	1.76–3.03	<0.001	2.18	1.66–2.87	<0.001	2.13	1.62–2.80	<0.001
Allergic rhinitis	1.42	1.30–1.55	<0.001	1.39	1.27–1.52	<0.001	1.33	1.21–1.45	<0.001
Conjunctivitis	1.29	1.15–1.45	<0.001	1.15	1.02–1.29	0.022	1.16	1.03–1.31	0.013
Urticaria	2.53	2.27–2.82	<0.001	2.37	2.13–2.64	<0.001	2.38	2.14–2.66	<0.001

a Log-rank test.

b Cox proportional hazards model.

c Competing events: death.

### Stratification analysis

In the age-stratified subgroup analyses, a decreasing risk was enhanced in patients younger than 40 years old, with a 20% decreased risk compared with non-NAFLD controls (aHR = 0.80, 95% CI 0.70–0.92) (**Table 4**). As for patients older than 40 years old, the significance of the NAFLD–atopic dermatitis association was not observed. For female NAFLD patients, the risk of atopic dermatitis was lower than in controls (aHR = 0.85, 95% CI 0.77–0.95). Moreover, a decreasing risk of atopic dermatitis was only observed in patients who were followed up for more than 3 years (aHR = 0.89, 95% CI 0.81–0.97). For those having less than 3 years of follow-up, the trend was statistically insignificant.

**Table 4:** Stratified analysis.

Variables	NAFLD vs. non-NAFLD controls (ref.)		
	aHR	95% CI	*p*-value
Age (years)			
≤40	0.80	0.70–0.92	<0.001
41–64	0.96	0.88–1.05	0.392
≥65	0.99	0.87–1.13	0.907
Gender			
Female	0.85	0.77–0.95	0.003
Male	0.98	0.90–1.07	0.624
Follow-up period below 3 years	1.02	0.93–1.12	0.701
Follow-up period 3 years above	0.89	0.81–0.97	0.010

Extraneous factors adjusted in the model contained all variables in **Table 2**.

aHR, adjusted hazard ratio.

## Discussion

In the current retrospective cohort study, we utilized a nationwide database to evaluate the risk of atopic dermatitis in people with NAFLD. Compared with people without NAFLD, a decreased risk of atopic dermatitis in NALFD patients had been observed (aHR = 0.93, 95% CI 0.87–0.98). The trend was especially presented in young NAFLD patients. In patients younger than 40 years old, a 20% decreased risk of atopic dermatitis was reported (aHR = 0.80, 95% CI 0.70–0.92).

NAFLD could influence multiple organ systems. Due to the influence of metabolic factors including obesity or diabetes, steatosis of hepatocytes was noted in NAFLD patients (16). In the pathogenesis of NAFLD, secretion of proinflammatory cytokines would lead to chronic inflammation, causing the potential association between NAFLD and various comorbidities. The common comorbidities of NAFLD include diabetes, cardiovascular diseases, and renal diseases (17). As for dermatological involvements, previous studies identified the association between NAFLD and psoriasis (13, 18). Previous articles suggested that elevated secretion of non-esterified fatty acids would be presented in liver tissue due to the risk factors of NAFLD, such as insulin resistance (19). Under this circumstance, oxidative stress could be possibly increased, leading to a subsequent inflammatory response (19, 20). A population-based study from South Korea hypothesized that through the excess accumulation of free fatty acids, chronic inflammation status could potentially lead to airway impairment and remodeling, attributed to the higher incidence of asthma in NAFLD patients (21).

The association between atopic dermatitis and NAFLD has been discussed in various previous studies. Fatty liver diseases have been regarded as one of the possible comorbidities of atopic dermatitis based on the available evidence to date (22). However, most studies were limited to a small sample size, a cross-sectional design, or animal models. In an animal study based on a mouse model, it was reported that in atopic dermatitis lesions, lipid metabolism would be influenced and the accumulation of cholesterol in hepatic tissues was noted (23). Another single-center study in Japan reported that the prevalence of fatty liver diseases was observed to be increased in pediatric populations (9). To the best of our knowledge, though an increased prevalence of atopic dermatitis was noted in previous literature, the actual mechanism regarding the interplay between NAFLD and atopic dermatitis is still unconfirmed. Moreover, previous studies did not focus on the longitudinal influence of NAFLD and the subsequent atopic dermatitis incidence. The current study provided real-world evidence regarding the knowledge gap of this issue and suggested that NAFLD patients were associated with a lower risk of atopic dermatitis. The results of our current study revealed that though the prevalence of NAFLD might be high in people with atopic dermatitis, the reversed association was not observed in longitudinal settings. To the best of our knowledge, the current mechanism hypotheses in the pathogenesis of NAFLD might not be sufficient to properly attribute the lower incidence of atopic dermatitis in the NAFLD group. However, we hypothesize that after NAFLD diagnosis, changes in the patients’ lifestyle could occur and therefore influence the incidence of atopic dermatitis. Given that we were not able to access lifestyle information and the exposure to allergens in patients, we believe that further large-scale studies might be necessary to evaluate the molecular mechanism in NAFLD patients and evaluate the potential role of lifestyle in the observed association. In the Cox regression analysis, it was presented that patients with coronary artery disease were associated with atopic dermatitis in the unadjusted model. However, after adjustment of covariates potentially influencing the incidence of atopic dermatitis, including age, sex, urbanization status, socioeconomic status, medical utilization status, and other comorbidities, the association became insignificant. The difference indicates that confounding biases could influence the interpretation of the results and should be prudently considered. A recent Mendelian randomization study also reported a non-significant causal association between atopic dermatitis and coronary heart diseases, which also corresponded to the observed trend in the adjusted model in the current study (24).

The limitations of this study should be stated. First, given that this study was based on a real-world and retrospective study setting, residual confounders could still exist even though we have tried our best to perform adjustments on potential confounders. The association observed in retrospective cohort studies should be carefully interpreted and should not be regarded as causation. Second, in the current study, the definition of diseases and other covariates was based on the ICD-9/10 codes, which could lead to potential misclassification bias. Previous studies had intended to validate the positive predictive values of ICD-9/10 of NAFLD and atopic dermatitis in claim-based datasets (25, 26). However, in our current study, misclassification bias could still exist due to the limitation of administrative codes. To the best of our knowledge, validation studies evaluating the positive predictive value of ICD codes of atopic dermatitis in Taiwanese population were not available. Although we have adopted in the current study the ICD coding algorithms utilized in a previous NHIRD study to define atopic dermatitis, some of the atopic dermatitis cases could still be potentially misclassified. Third, since environmental, lifestyle, and genetic information was not available in the NHIRD, we were not able to set these factors as covariates to evaluate the outcomes. Fourth, since the information on the widely used NAFLD severity indicators such as NAFLD fibrosis score or steatosis, activity, and fibrosis (SAF) score was not available in the NHIRD, we were not able to precisely define the severity of NAFLD (27, 28). Therefore, stratification analysis based on NAFLD severity was not available in the current study. Fifth, datasets from the NHIRD of Taiwan might not be generalizable to other populations with cultural, genetic, or environmental differences. The aforementioned limitations should be noted by the authors, and the results of the current study should be prudently interpreted.

As a conclusion, people with NAFLD were not associated with an increased risk of atopic dermatitis. Conversely, a 0.93-fold risk was noted in NAFLD patients, compared with NAFLD-free controls. Future studies are warranted to evaluate further the mechanism regarding the interplay between the inflammatory mechanisms of NAFLD and atopic dermatitis.

## Data availability statement

The original contributions presented in the study are included in the article/**Supplementary Material**. Further inquiries can be directed to the corresponding author.

## Ethics statement

The studies involving human participants were reviewed and approved by the Central Regional Research Ethics Committee of China Medical University, Taiwan. Written informed consent for participation was not required for this study in accordance with the national legislation and the institutional requirements.

## Author contributions

Study conception and design: S-YG, K-HH, C-HH, T-HT, YY, and C-YL. Data acquisition: T-HT, K-HH, and C-YL. Data analysis and demonstration: S-YG, T-HT, and C-YL. Original draft preparation: S-YG, T-HT, K-HH, C-HH, YY, and C-YL. All authors contributed to the article and approved the submitted version.

## References

[1] Neuschwander-Tetri BA. Non-alcoholic fatty liver disease. BMC Med (2017) 15(1):45. doi: 10.1186/s12916-017-0806-8 28241825 PMC5330146

[2] Younossi ZM Koenig AB Abdelatif D Fazel Y Henry L Wymer M. Global epidemiology of nonalcoholic fatty liver disease-Meta-analytic assessment of prevalence, incidence, and outcomes. Hepatology (2016) 64(1):73–84. doi: 10.1002/hep.28431 26707365

[3] Johnston MP Patel J Byrne CD. Causes of mortality in non-alcoholic fatty liver disease (NAFLD) and alcohol related fatty liver disease (AFLD). Curr Pharm Des (2020) 26(10):1079–92. doi: 10.2174/1381612826666200128094231 32003662

[4] Glass LM Hunt CM Fuchs M Su GL. Comorbidities and nonalcoholic fatty liver disease: the chicken, the egg, or both? Fed Pract (2019) 36(2):64–71. 30867626 PMC6411365

[5] Stander S. Atopic dermatitis. N Engl J Med (2021) 384(12):1136–43. doi: 10.1056/NEJMra2023911 33761208

[6] Nutten S. Atopic dermatitis: global epidemiology and risk factors. Ann Nutr Metab (2015) 66(Suppl 1):8–16. doi: 10.1159/000370220 25925336

[7] Yong SB Gau SY Guo YC Wei JC. Allergy from perspective of environmental pollution effects: from an aspect of atopic dermatitis, immune system, and atmospheric hazards-a narrative review of current evidences. Environ Sci pollut Res Int (2022) 29(38):57091–57101. doi: 10.1007/s11356-022-21582-3 35759095

[8] Dai YX Tai YH Chang YT Chen TJ Chen MH. Bidirectional association between psoriasis and atopic dermatitis: A nationwide population-based cohort study. Dermatology (2021) 237(4):521–7. doi: 10.1159/000514581 33735855

[9] Kimata H. Prevalence of fatty liver in non-obese Japanese children with atopic dermatitis. Indian Pediatr (2005) 42(6):587–93. 15995275

[10] Kimata H. Increased incidence of fatty liver in non-obese Japanese children under 1 year of age with or without atopic dermatitis. Public Health (2006) 120(2):176–8. doi: 10.1016/j.puhe.2005.02.006 16337981

[11] Hsieh CY Su CC Shao SC Sung SF Lin SJ Kao Yang YH. Taiwan's National Health Insurance Research Database: past and future. Clin Epidemiol (2019) 11:349–58. doi: 10.2147/CLEP.S196293 PMC6509937 31118821

[12] Lin LY Warren-Gash C Smeeth L Chen PC. Data resource profile: the National Health Insurance Research Database (NHIRD). Epidemiol Health (2018) 40:e2018062. doi: 10.4178/epih.e2018062 30727703 PMC6367203

[13] Gau SY Huang KH Lee CH Kuan YH Tsai TH Lee CY. Bidirectional association between psoriasis and nonalcoholic fatty liver disease: real-world evidence from two longitudinal cohort studies. Front Immunol (2022) 13:840106. doi: 10.3389/fimmu.2022.840106 35251036 PMC8889012

[14] Lin TL Wu CY Yen JJ Juan CK Chang YL Ho HJ. Fracture risks in patients with atopic dermatitis: A nationwide matched cohort study. Ann Allergy Asthma Immunol (2021) 127(6):667–673.e662. doi: 10.1016/j.anai.2021.09.004 34537357

[15] Charlson ME Carrozzino D Guidi J Patierno C. Charlson comorbidity index: A critical review of clinimetric properties. Psychother Psychosom (2022) 91(1):8–35. doi: 10.1159/000521288 34991091

[16] Powell EE Wong VW Rinella M. Non-alcoholic fatty liver disease. Lancet (2021) 397(10290):2212–24. doi: 10.1016/S0140-6736(20)32511-3 33894145

[17] Rosato V Masarone M Dallio M Federico A Aglitti A Persico M. NAFLD and extra-hepatic comorbidities: current evidence on a multi-organ metabolic syndrome. Int J Environ Res Public Health (2019) 16(18). doi: 10.3390/ijerph16183415 PMC6765902 31540048

[18] Ruan Z Lu T Chen Y Yuan M Yu H Liu R. Association between psoriasis and nonalcoholic fatty liver disease among outpatient US adults. JAMA Dermatol (2022) 158(7):745–53. doi: 10.1001/jamadermatol.2022.1609 PMC9134040 35612851

[19] Mantovani A Gisondi P Lonardo A Targher G. Relationship between non-alcoholic fatty liver disease and psoriasis: A novel hepato-dermal axis? Int J Mol Sci (2016) 17(2):217. doi: 10.3390/ijms17020217 26861300 PMC4783949

[20] Hunter CA Jones SA. IL-6 as a keystone cytokine in health and disease. Nat Immunol (2015) 16(5):448–57. doi: 10.1038/ni.3153 25898198

[21] Roh JH Lee H Yun-Jeong B Park CS Kim HJ Yoon SY. A nationwide survey of the association between nonalcoholic fatty liver disease and the incidence of asthma in Korean adults. PloS One (2022) 17(1):e0262715. doi: 10.1371/journal.pone.0262715 35061826 PMC8782316

[22] Silverberg JI Silverberg NB. Atopic dermatitis: update on pathogenesis and comorbidities. Curr Dermatol Rep (2012) 1(4):168–78. doi: 10.1007/s13671-012-0021-y

[23] Seino S Tanaka Y Honma T Yanaka M Sato K Shinohara N. et al: Atopic dermatitis causes lipid accumulation in the liver of NC/Nga mouse. J Clin Biochem Nutr (2012) 50(2):152–7. doi: 10.3164/jcbn.11-29 PMC3303478 22448097

[24] Huang J Gui Y Wu J Xie Y. Investigating the association of atopic dermatitis with ischemic stroke and coronary heart disease: A mendelian randomization study. Front Genet (2022) 13:956850. doi: 10.3389/fgene.2022.956850 36110212 PMC9468876

[25] Corey KE Kartoun U Zheng H Shaw SY. Development and validation of an algorithm to identify nonalcoholic fatty liver disease in the electronic medical record. Dig Dis Sci (2016) 61(3):913–9. doi: 10.1007/s10620-015-3952-x PMC4761309 26537487

[26] Hsu DY Dalal P Sable KA Voruganti N Nardone B West DP. Validation of International Classification of Disease Ninth Revision codes for atopic dermatitis. Allergy (2017) 72(7):1091–5. doi: 10.1111/all.13113 PMC5461190 27997983

[27] Nascimbeni F Bedossa P Fedchuk L Pais R Charlotte F Lebray P. Clinical validation of the FLIP algorithm and the SAF score in patients with non-alcoholic fatty liver disease. J Hepatol (2020) 72(5):828–38. doi: 10.1016/j.jhep.2019.12.008 31862486

[28] Treeprasertsuk S Bjornsson E Enders F Suwanwalaikorn S Lindor KD. NAFLD fibrosis score: a prognostic predictor for mortality and liver complications among NAFLD patients. World J Gastroenterol (2013) 19(8):1219–29. doi: 10.3748/wjg.v19.i8.1219 PMC3587478 23482703

