# Supplementary material for: The association between non-alcoholic fatty liver disease and atopic dermatitis: a population-based cohort study
Source: Front Immunol. 2023 Aug 18;14:1171804. doi: 10.3389/fimmu.2023.1171804 (PMC10471967; doi:10.3389/fimmu.2023.1171804)
Supplement: Supplementary file 1 [file Table_1.docx]

**Supplementary File**

1. ICD codes utilized in the current study

| Diseases | ICD-9 | ICD10 |
| --- | --- | --- |
| non-alcoholic fatty liver diseases | 571.8 | K76.0; K75.81 |
| atopic dermatitis | 691.8 | L20 |
| liver fibrosis and cirrhosis | 571.2; 571.6 | K74 |
| liver cancer | 155.0 | C22 |
| hypertension | 401-405 | I10 |
| diabetes | 250 | E10-E13 |
| inflammatory bowel diseases | 555; 556 | K50; K51 |
| hyperlipidemia | 272 | E78.2-E78.4 |
| myocardial infarction | 410 | I21 |
| coronary artery disease | 411-414 | I25.1 |
| chronic kidney disease | 585 | N18 |
| obesity | 278.0 | E66 |
| alcoholism | 303.0; 303.9 | F10.20 |
| psoriasis | 696.0; 696.1 | L40 |
| asthma | 493 | J45 |
| allergic rhinitis | 477.9 | J30.1 |
| conjunctivitis | 372.30 | H10 |
| urticaria | 708.9 | L50 |
